# Skeletal Muscle Cell Induction from Pluripotent Stem Cells

**DOI:** 10.1155/2017/1376151

**Published:** 2017-04-26

**Authors:** Yusaku Kodaka, Gemachu Rabu, Atsushi Asakura

**Affiliations:** ^1^Stem Cell Institute, University of Minnesota Medical School, Minneapolis, MN 55455, USA; ^2^Paul and Sheila Wellstone Muscular Dystrophy Center, University of Minnesota Medical School, Minneapolis, MN 55455, USA; ^3^Department of Neurology, University of Minnesota Medical School, Minneapolis, MN 55455, USA

## Abstract

Embryonic stem cells (ESCs) and induced pluripotent stem cells (iPSCs) have the potential to differentiate into various types of cells including skeletal muscle cells. The approach of converting ESCs/iPSCs into skeletal muscle cells offers hope for patients afflicted with the skeletal muscle diseases such as the Duchenne muscular dystrophy (DMD). Patient-derived iPSCs are an especially ideal cell source to obtain an unlimited number of myogenic cells that escape immune rejection after engraftment. Currently, there are several approaches to induce differentiation of ESCs and iPSCs to skeletal muscle. A key to the generation of skeletal muscle cells from ESCs/iPSCs is the mimicking of embryonic mesodermal induction followed by myogenic induction. Thus, current approaches of skeletal muscle cell induction of ESCs/iPSCs utilize techniques including overexpression of myogenic transcription factors such as MyoD or Pax3, using small molecules to induce mesodermal cells followed by myogenic progenitor cells, and utilizing epigenetic myogenic memory existing in muscle cell-derived iPSCs. This review summarizes the current methods used in myogenic differentiation and highlights areas of recent improvement.

## 1. Introduction

Duchenne muscular dystrophy (DMD) is a genetic disease affecting approximately 1 in 3500 male live births [1]. It results in progressive degeneration of skeletal muscle causing complete paralysis, respiratory and cardiac complications, and ultimately death. Normal symptoms include the delay of motor milestones including the ability to sit and stand independently. DMD is caused by an absence of functional dystrophin protein and skeletal muscle stem cells, as well as the exhaustion of satellite cells following many rounds of muscle degeneration and regeneration [2]. The dystrophin gene is primarily responsible for connecting and maintaining the stability of the cytoskeleton of muscle fibers during contraction and relaxation. Despite the low frequency of occurrence, this disease is incurable and will cause debilitation of the muscle and eventual death in 20 to 30 year olds with recessive X-linked form of muscular dystrophy. Although there are no current treatments developed for DMD, there are several experimental therapies such as stem cell therapies.

Skeletal muscle is known to be a regenerative tissue in the body. This muscle regeneration is mediated by muscle satellite cells, a stem cell population for skeletal muscle [3, 4]. Although satellite cells exhibit some multipotential differentiation capabilities [5], their primary differentiation fate is skeletal muscle cells in normal muscle regeneration. Ex vivo expanded satellite cell-derived myoblasts can be integrated into muscle fibers following injection into damaged muscle, acting as a proof-of-concept of myoblast-mediated cell therapy for muscular dystrophies [6–9]. However, severe limitations exist in relation to human therapy. The number of available satellite cells or myoblasts from human biopsies is limited. In addition, the poor cell survival and low contribution of transplanted cells have hindered practical application in patients [6, 8, 9]. Human-induced pluripotent stem cells (hiPSCs) are adult cells that have been genetically reprogrammed to an embryonic stem cell- (ESC-) like state by being forced to express genes and factors important for maintaining the defining properties of ESCs. hiPSCs can be generated from a wide variety of somatic cells [10, 11]. They have the ability to self-renew and successfully turn into any type of cells. With their ability to capture genetic diversity of DMD in an accessible culture system, hiPSCs represent an attractive source for generating myogenic cells for drug screening.

The ESC/iPSC differentiation follows the steps of embryonic development. The origin of skeletal muscle precursor cells comes from the mesodermal lineage, which give rise to skeletal muscle, cardiac muscle, bone, and blood cells. Mesoderm subsequently undergoes unsegmented presomitic mesoderm followed by segmented compartments termed somites from anterior to caudal direction. Dermomyotome is an epithelial cell layer making up the dorsal part of the somite underneath the ectoderm. Dermomyotome expresses Pax3 and Pax7 and gives rise to dermis, skeletal muscle cells, endothelial cells, and vascular smooth muscle [12]. Dermomyotome also serves as a tissue for secreted signaling molecules to the neural tube, notochord, and sclerotome [13, 14]. Upon signals from the neural tube and notochord, the dorsomedial lip of dermomyotome initiates and expresses skeletal muscle-specific transcription factors such as MyoD and Myf5 to differentiate into myogenic cells termed myoblasts. Myoblasts then migrate beneath the dermomyotome to form myotome. Eventually, these myoblasts fuse with each other to form embryonic muscle fibers. ESCs/iPSCs mimic these steps toward differentiation of skeletal muscle cells. Many studies utilize methods of overexpression of muscle-related transcription factors such as MyoD or Pax3 [15], or the addition of small molecules which activate or inhibit myogenic signaling during development. Several studies show that iPSCs retain a bias to form their cell type of origin due to an epigenetic memory [16–19], although other papers indicate that such epigenetic memory is erased during the reprogramming processes [20–22]. Therefore, this phenomenon is not completely understood at the moment. In light of these developments, we have recently established mouse myoblast-derived iPSCs capable of unlimited expansion [23]. Our data demonstrates that these iPSCs show higher myogenic differentiation potential compared to fibroblast-derived iPSCs. Thus, myogenic precursor cells generated from human myoblast-derived iPSCs expanded ex vivo should provide an attractive cell source for DMD therapy. However, since DMD is a systemic muscle disease, systemic delivery of myoblasts needs to be established for efficient cell-based therapy.

## 2. Myogenic Master Transcription Factors for Skeletal Muscle Development (Figure 1)

**Figure 1:**
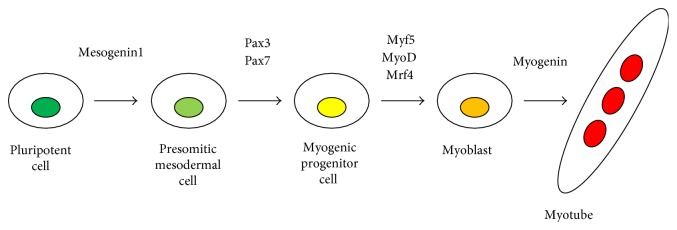
Hierarchal master transcription factor cascade for myogenesis. For myogenic differentiation during early embryogenesis, Mesogenin1 works as a master regulator for unsegmented presomitic mesoderm formation. Then, segmented somites are formed. Pax3 and Pax7 are activated in presomitic mesoderm, which generates somite-derived dermomyotome. Pax3 and Pax7 then work as master regulators for myogenic progenitor cell induction. Finally, MyoD and Myf5 are upregulated in the dorsomedial lip of dermomyotome and function as master regulators for myogenic specification to generate myoblasts. Eventually, myoblasts stop cell proliferation and express myogenin, which induces terminal differentiation of myoblasts to form multinucleated myotubes.

During developmental myogenesis, presomitic mesoderm is first formed by Mesogenin1 upregulation, which is a master regulator of presomitic mesoderm [24]. Then, the paired box transcription factor *Pax3* gene begins to be expressed from presomitic mesoderm to dermomyotome [25]. Following *Pax3* expression, *Pax7* is also expressed in the dermomyotome [26], and then *Myf5* and *MyoD*, skeletal muscle-specific transcription factor genes, begin to be expressed in the dorsomedial lip of the dermomyotome in order to give rise to myoblasts which migrate beneath the dermomyotome to form the myotome. Subsequently, *Mrf4* and *Myogenin*, other skeletal muscle-specific transcription factor genes, followed by skeletal muscle structural genes such as myosin heavy chain (MyHC), are expressed in the myotome for myogenic terminal differentiation (Figure 1) [27, 28]. *Pax3* directly and indirectly regulates *Myf5* expression in order to induce myotomal cells. Dorsal neural tube-derived Wnt proteins and floor plate cells in neural tube and notochord-derived sonic hedgehog (Shh) positively regulate myotome formation [13, 29]. Neural crest cells migrating from dorsal neural tubes are also involved in myotome formation: Migrating neural crest cells come across the dorsomedial lip of the dermomyotome, and neural crest cell-expressing Delta1 is transiently able to activate Notch1 in the dermomyotome, resulting in conversion of Pax3/7(+) myogenic progenitor cells into MyoD/Myf5(+) myotomal myoblasts [30, 31]. By contrast, bone morphogenetic proteins (BMPs) secreted from lateral plate mesoderm are a negative regulator for the myotome formation by maintaining Pax3/Pax7(+) myogenic progenitor cells [29, 32]. Pax3 also regulates cell migration of myogenic progenitor cells from ventrolateral lip of dermomyotome to the limb bud [33]. *Pax3* mutant mice lack limb muscle but trunk muscle development is relatively normal [34]. *Pax3/Pax7* double knockout mice display failed generation of myogenic cells, suggesting that *Pax3* and *Pax7* are critical for proper embryonic myogenesis [35]. Therefore, both *Pax3* and *Pax7* are also considered master transcription factors for the specification of myogenic progenitor cells. Importantly, MyoD was identified as the first master transcription factor for myogenic specification since MyoD is directly able to reprogram nonmuscle cell type to myogenic lineage when overexpressed [36–38]. In addition, genetic ablation of MyoD family gene(s) via a homologous gene recombination technique causes severe myogenic developmental or regeneration defects [39–45]. Finally, genetic ablation of combinatory MyoD family genes demonstrates that *MyoD^−/−^:Myf5^−/−^:MRF4^−/−^* mice do not form any skeletal muscle during embryogenesis, indicating the essential roles in skeletal muscle development of MyoD family genes [28, 46]. It was proven that Pax3 also possesses myogenic specification capability since ectopic expression of Pax3 is sufficient to induce myogenic programs in both paraxial and lateral plate mesoderm as well as in the neural tube during chicken embryogenesis [47]. In addition, genetic ablation of Pax3 and Myf5 display complete defects of body skeletal muscle formation during mouse embryogenesis [48]. Finally, overexpression of Pax7 can convert CD45(+)Sca-1(+) hematopoietic cells into skeletal muscle cells [49]. From these notions, overexpression of myogenic master transcription factors such as MyoD or Pax3 has become the major strategy for myogenic induction in nonmuscle cells, including ES/iPSCs.

## 3. Overexpression Approaches of Myogenic Master Transcription Factors in ESCs/iPSCs

The overexpression of *MyoD* approach to induce myogenic cells from mESCs was first described by Dekel et al. in 1992. This has been a standard approach for the myogenic induction from pluripotent stem cells (Table 1). Ozasa et al. first utilized Tet-Off systems for *MyoD* overexpression in mESCs and showed desmin(+) and MyHC(+) myotubes in vitro [50]. Warren et al. transfected synthetic *MyoD* mRNA in to hiPSCs for 3 days, which resulted in myogenic differentiation (around 40%) with expression of myogenin and MyHC [51]. Tanaka et al. utilized a *PiggyBac* transposon system to overexpress *MyoD* in hiPSCs. The *PiggyBac* transposon system allows cDNAs to stably integrate into the genome for efficient gene expression. After integration, around 70 to 90% of myogenic cells were induced in hiPSC cultures within 5 days [52]. This study also utilized Miyoshi myopathy patient-derived hiPSCs for the MyoD-mediated myogenic differentiation. Miyoshi myopathy is a congenital distal myopathy caused by defective muscle membrane repair due to mutations in *dysferlin* gene. The patient-derived hiPSC-myogenic cells will be able to provide the opportunity for therapeutic drug screening. Abujarour et al. also established a model of patient-derived skeletal muscle cells which express NCAM, myogenin, and MyHC by doxycycline-inducible overexpression of *MyoD* in DMD patient-derived hiPSCs [53]. Interestingly, MyoD-induced iPSCs also showed suppression of pluripotent genes such as Nanog and a transient increase in the gene expression levels of *T (Brachyury T)*, *Pax3*, and *Pax7*, which belong to paraxial mesodermal/myogenic progenitor genes, upstream genes of myogenesis. It is possible that low levels of MyoD activity in hiPSCs may initially suppress their pluripotent state while failing to induce myogenic programs, which may result in transient paraxial mesodermal induction. Supporting this idea, BAF60C, a SWI/SNF component that is involved in chromatin remodeling and binds to MyoD, is required to induce full myogenic program in MyoD-overexpressing hESCs [54]. Overexpression of MyoD alone in hESC can only induce some paraxial mesodermal genes such as *Brachyury T*, *mesogenin*, and *Mesp1* but not myogenic genes. Co-overexpression of MyoD and BAF60C was now able to induce myogenic program but not paraxial mesodermal gene expression, indicating that there are different epigenetic landscapes between pluripotent ESCs/iPSCs and differentiating ESC/iPSCs in which MyoD is more accessible to DNA targets than those in pluripotent cells. The authors then argued that without specific chromatin modifiers, only committed cells give rise to myogenic cells by MyoD. These results strongly indicate that nuclear landscapes are important for cell homogeneity for the specific cell differentiation in ESC/iPSC cultures. Similar observations were seen in overexpression of *MyoD* in P19 embryonal carcinoma stem cells, which can induce paraxial mesodermal genes including Meox1, Pax3, Pax7, Six1, and Eya2 followed by muscle-specific genes. However, these MyoD-induced paraxial mesodermal genes were mediated by direct MyoD binding to their regulatory regions, which was proven by chromatin immunoprecipitation (ChIP) assays, indicating the novel role for MyoD in paraxial mesodermal cell induction [55].

hESCs/iPSCs have been differentiated into myofibers by overexpression of *MyoD*, and this method is considered an excellent in vitro model for human skeletal muscle diseases for muscle functional tests, therapeutic drug screening, and genetic corrections such as exon skipping and DNA editing. Shoji et al. have shown that DMD patient-derived iPSCs were used for myogenic differentiation via *PiggyBac*-mediated MyoD overexpression. These myogenic cells were treated with morpholinos for exon-skipping strategies for *dystrophin* gene correction and showed muscle functional improvement [56]. Li et al. have shown that patient-derived hiPSC gene correction by TALEN and CRISPR-Cas9 systems, and these genetically corrected hiPSCs were used for myogenic differentiation via overexpression of *MyoD* [57]. This work also revealed that the TALEN and CRISPR-Cas9-mediated exon 44 knock-in approach in the *dystrophin* gene has high efficiency in gene-editing methods for DMD patient-derived cells in which the exon 44 is missing in the genome.

Along this line of the strategy, Darabi et al. first performed overexpression of *Pax3* gene, which can be activated by treatment with doxycycline in mESCs, and showed efficient induction of MyoD/Myf5(+) skeletal myoblasts in EB cultures [15]. Upon removing doxycycline, these myogenic cells underwent MyHC(+) myotubes. However, teratoma formation was observed after EB cell transplantation into cardiotoxin-injured regenerating skeletal muscle in *Rag2^−/−^:γC^−/−^* immunodeficient mice [15]. This indicates that myogenic cell cultures induced by *Pax3* in mESCs still contain some undifferentiated cells which gave rise to teratomas. To overcome this problem, the same authors separated paraxial mesodermal cells from Pax3-induced EB cells by FACS using antibodies against cell surface markers as PDGFR*α*(+)Flk-1(−) cell populations. After cell sorting, isolated Pax3-induced paraxial mesodermal cells were successfully engrafted and contributed to regenerating muscle in *mdx:Rag2^−/−^:γC^−/−^* DMD model immunodeficient mice without any teratoma formations. Darabi et al. also showed successful myogenic induction in mESCs and hES/iPSCs by overexpression of Pax7 [58, 59]. *Pax3* and *Pax7* are not only expressed in myogenic progenitor cells. They are also expressed in neural tube and neural crest cell-derived cells including a part of cardiac cell types in developmental stage, suggesting that further purification to skeletal muscle cell lineage is crucial for therapeutic applications for muscle diseases including DMD.

Taken together, overexpression of myogenic master transcription factors such as MyoD or Pax3/Pax7 is an excellent strategy for myogenic induction in hESCs and hiPSCs, which can be utilized for in vitro muscle disease models for their functional test and drug screening. However, for the safe stem cell therapy, it is essential to maintain the good cellular and genetic qualities of hESC/hiPSC-derived myogenic cells before transplantation. Therefore, random integration sites of overexpression vectors for myogenic master transcription factors and inappropriate expression control of these transgenes may diminish the safety of using these induced myogenic cells for therapeutic stem cell transplantation.

## 4. Supplement with Defined Factors for Myogenic Induction in ESCs/iPSCs

**Figure 2:**
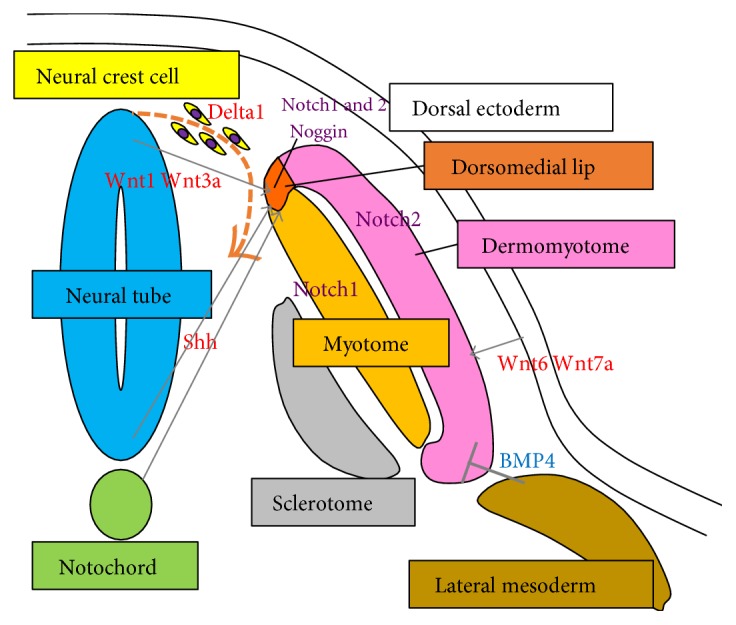
Positive and negative signals from surrounding tissues for embryonic myogenesis. Dermomyotome receives positive (Shh, Wnt1, Wnt3a, Wnt6, Wnt7a, Delta1, and Noggin) and negative (BMP4) signals from surrounding tissues (dorsal neutral tube, floor plate, notochord, dorsal ectoderm, and lateral mesoderm) to form myotomes. This occurs at the Notch1/2-positive dorsomedial lip of dermomyotome.

Stepwise induction protocols utilizing small molecules and growth factors have been established as alternative myogenic induction approaches and a more applicable method for therapeutic situations. As described above, during embryonic myogenesis, somites and dermomyotomes receive secreted signals such as Wnts, Notch ligands, Shh, FGF, BMP, and retinoic acid (RA) with morphogen gradients from surrounding tissues in order to induce the formation of myogenic cells (Figure 2). The canonical Wnt signaling pathway has been shown to play essential roles in the development of myogenesis. In mouse embryogenesis, Wnt1 and Wnt3a secreted from the dorsal neural tube can promote myogenic differentiation of dorsomedial dermomyotome via activation of Myf5 [31, 32, 60]. Wnt3a is able to stabilize *β*-catenin which associates with TCF/LEF transcription factors that bind to the enhancer region of Myf5 during myogenesis [61]. Other Wnt proteins, Wnt6 and Wnt7a, which emerge from the surface ectoderm, induce MyoD [62]. BMP functions as an inhibitor of myogenesis by suppression of some myogenic gene expressions. In the lateral mesoderm, BMP4 is able to increase Pax3 expression which delays Myf5 expression in order to maintain an undifferentiated myogenic progenitor state [63]. Therefore, Wnts and BMPs regulate myogenic development by antagonizing each other for myogenic transcription factor gene expression [64, 65]. Wnt also induces Noggin expression to antagonize BMP signals in the dorsomedial lip of the dermomyotome [66]. In this region, MyoD expression level is increased, which causes myotome formation. Notch signaling plays essential roles for cell-cell communication to specify the different cells in developmental stages. During myotome formation, Notch is expressed in dermomyotome, and Notch1 and Notch2 are expressed in dorsomedial lip of dermomyotome. Delta1, a Notch ligand, is expressed in neural crest cells which transiently interact with myogenic progenitor cells in dorsomedial lip of dermomyotome via Notch1 and 2. This contact induces expression of the Myf5 or MyoD gene in the myogenic progenitor cells followed by myotome formation. The loss of function of Delta1 in the neural crest displays delaying skeletal muscle formation [67]. Knockdown of Notch genes or use of a dominant-negative form of mastermind, a Notch transcriptional coactivator, clearly shows dramatically decrease of Myf5 and MyHC(+) myogenic cells. Interestingly, induction of Notch intracellular domain (NICD), a constitutive active form of Notch, can promote myogenesis, while continuous expression of NICD prevents terminal differentiation. Taken together, transient and timely activation of Notch is crucial for myotome formation from dermomyotome [30].

Current studies for myogenic differentiation of ESCs/iPSCs have utilized supplementation with some growth factors and small molecules, which would mimic the myogenic development described above in combination with embryoid body (EB) aggregation and FACS separation of mesodermal cells (Table 2). To induce paraxial mesoderm cells from mESCs, Sakurai et al. utilized BMP4 in serum-free cultures [68]. Three days after treatment with BMP4, mESCs could be differentiated into primitive streak mesodermal-like cells, but the continuous treatment with BMP4 turned the ESCs into osteogenic cells. Therefore, they used LiCl after treatment with BMP4 to enhance Wnt signaling, which is able to induce myogenic differentiation. After treatment with LiCl, PDGFR*α*(+) E-cadherin(−) paraxial mesodermal cells were sorted by FACS. These sorted cells were cultured with IGF, HGF, and FGF for two weeks in order to induce myogenic differentiation. Hwang et al. have shown that treatment with Wnt3a efficiently promotes skeletal muscle differentiation of hESCs [69]. hESCs were cultured to form EB for 9 days followed by differentiation of EBs for additional 7 days, and then PDGFR*α*(+) cells were sorted by FACS. These PDGFR*α*(+) cells were cultured with Wnt3a for additional 14 days. Consequently, these Wnt3a-treated cells display significantly increased myogenic transcription factors and structural proteins at both mRNA and protein levels. An interesting approach to identify key molecules that induce myogenic cells was reported by Xu et al. [70]. They utilized reporter systems in zebrafish embryos to display myogenic progenitor cell induction and myogenic differentiation in order to identify small compounds for myogenic induction. *Myf5-GFP* marks myogenic progenitor cells, while *myosin light polypeptide 2* (*mylz2*)-*mCherry* marks terminally differentiated muscle cells. They found that a mixed cocktail containing GSK3*β* inhibitor, bFGF, and forskolin has the potential to induce robust myogenic induction in hiPSCs. GSK3*β* inhibitors act as a canonical Wnt signaling activator via stabilizing *β*-catenin protein, which is crucial for inducing mesodermal cells. Forskolin activates adenylyl cyclase, which then stimulates cAMP signaling. cAMP response element-binding protein (CREB) is able to stimulate cell proliferation of primary myoblasts in vitro, suggesting that the forskolin-cAMP-CREB pathway may help myogenic cell expansion [71], However the precise mechanisms for CREB-mediated myogenic cell expansion remain unclear. The adenylyl cyclase signaling cascade leads to CREB activation [71]. During embryogenesis, phosphorylated CREB has been found at dorsal somite and dermomyotome. CREB gene knockout mice display significantly decreased Myf5 and MyoD expressions in myotomes. While activation of Wnt1 or Wnt7a promotes Pax3, Myf5, and MyoD expressions, inhibition of CREB eliminates these Wnt-mediated myogenic gene expressions without altering the Wnt canonical pathway, suggesting that CREB-induced myogenic activation may be mediated through noncanonical Wnt pathways. Several groups also utilized GSK3*β* inhibitors for inducing mesodermal cells from ESCs and iPSCs [72, 73]. These mesodermal cell-like cells were expanded by treatment with bFGF, and then ITS (insulin/transferrin/selenite) or N2 medium were used to induce myogenic differentiation. Finally, bFGF is a stimulator for myogenic cell proliferation. Caron et al. demonstrated that hESCs treated with GSK3*β* inhibitor, ascorbic acid, Alk5 inhibitor, dexamethasone, EGF, and insulin generated around 80% of Pax3(+) myogenic precursor cells in 10 days [74]. Treatment with SB431542, an inhibitor of Alk4, 5, and 7, PDGF, bFGF, oncostatin, and IGF was able to induce these Pax3(+) myogenic precursor cells into around 50–60% of MyoD(+) myoblasts in an additional 8 days. For the final step, treatment with insulin, necrosulfonamide, an inhibitor of necrosis, oncostatin, and ascorbic acid was able to induce these myoblasts into myotubes in an additional 8 days. Importantly, the same authors utilized ESCs from human facioscapulohumeral muscular dystrophy (FSHD) to demonstrate the myogenic characterization after myogenic induction by using the protocol described above. Hosoyama et al. have shown that hESCs/iPSCs with high concentrations of bFGF and EGF in combination with cell aggregation, termed EZ spheres, efficiently give rise to myogenic cells [75]. After 6-week culture, around 40–50% of cells expressed Pax7, MyoD, or myogenin. However, the authors also showed that EZ spheres included around 30% of Tuj1(+) neural cells. Therefore, the authors discussed the utilization of molecules for activation of mesodermal and myogenic signaling pathways such as BMPs and Wnts.

Taken together, it is likely that the induced cell populations from ESCs/iPSCs may contain other cell types such as neural cells or cardiac cells because neural cells share similar transcription factor gene expression with myogenic cells such as Pax3, and cardiac cells also develop from mesodermal cells. To overcome this limitation, Chal et al. treated ESCs/iPSCs with BMP4 inhibitor, which prevents ESCs/iPSCs from differentiating into lateral mesodermal cells [76, 77]. To identify what genes are involved in myogenic differentiation in vivo, they performed a microarray analysis which compared samples of dissected fragments in mouse embryos, which are able to separate tail bud, presomitic mesoderm, and somite regions. From microarray data, the authors focused on *Mesogenin1* (*Msgn1*) and *Pax3* genes. Importantly, they utilized three lineage tracing reporters, *Msgn1-repV* (*Mesogenin1-Venus*) marking posterior somitic mesoderm, *Pax3-GFP* marking anterior somitic mesoderm and myogenic cells, and *Myog-repV* (*Myogenin-Venus*) marking differentiated myocytes, allowing the authors to readily detect different differentiation stages during ESC/iPSC cultures. Treatment with GSK3*β* inhibitors and then BMP inhibitors in ESC cultures induced Msgn1(+) somitic mesoderm with 45 to 65% efficiencies, Pax3(+) anterior somitic mesoderm with 30 to 50% efficiencies, and myogenin(+) myogenic cells with 25 to 30% efficiencies. Furthermore, the authors examined differentiation of *mdx* ESCs into skeletal muscle cells and revealed abnormal branching myofibers. Current protocols were also published and described more details for hiPSC differentiation [77].

## 5. Induction of Skeletal Muscle Cells from iPSC-Derived Mesoangioblast-Like Cells

Some nonmuscle cell populations such as mesoangioblasts have the potential to differentiate into skeletal muscle [6]. Mesoangioblasts were originally isolated from embryonic mouse dorsal aorta as vessel-associated pericyte-like cells, which have the ability to differentiate into a myogenic lineage in vitro and in vivo [6, 78]. Mesoangioblasts possess an advantage for the clinical cell-based treatment because they can be injected through an intra-arterial route to systemically deliver cells, which is crucial for therapeutic cell transplantation for muscular dystrophies [79]. Tedesco et al. successfully generated human iPSC-derived mesoangioblast-like stem/progenitor cells called HIDEMs by stepwise protocols without FACS sorting [80, 81]. They displayed similar gene expression profiles as embryonic mesoangioblasts. However, HIDEMs do not spontaneously differentiate into skeletal muscle cells, and thus, the authors utilized overexpression of *MyoD* to differentiate into skeletal muscle cells. Similar to mesoangioblasts, HIDEM-derived myogenic cells could be delivered to injured muscle via intramuscular and intra-arterial routes. Furthermore, HIDEMs have been generated from hiPSCs derived from limb-girdle muscular dystrophy (LGMD) type 2D patients and used for gene correction and cell transplantation experiments for the potential therapeutic application.

## 6. Enrichment of ESC/iPSC-Derived Myogenic Precursor Cells

Myogenic precursor cells derived from ESCs/iPSCs by various methods may contain nonmuscle cells. Therefore, further purification is mandatory for therapeutic applications. Barberi et al. isolated CD73(+) multipotent mesenchymal precursor cells from hESCs by FACS, and these cells underwent differentiation into fat, cartilage, bone, and skeletal muscle cells [82]. Barberi et al. also demonstrated that hESCs cultured on OP9 stroma cells generated around 5% of CD73(+) adult mesenchymal stem cell-like cells [83]. After FACS, these CD73(+) mesenchymal stem cell-like cells were cultured with ITS medium for 4 weeks and then gave rise to NCAM(+) myogenic cells. After FACS sorting, these NCAM(+) myogenic cells were purified by FACS and transplanted into immunodeficient mice to show their myogenic contribution to regenerating muscle.

It has been shown that many genes are associated with myogenesis. In addition, exhaustive analysis, such as microarray, RNA-seq, and single cell RNA-seq supplies much gene information in many different stages. Chal et al. showed key signaling factors by microarray from presomitic somite, somite, and tail bud cells [76]. They found that initial Wnt signaling has important roles for somite differentiation. Furthermore, mapping differentiated hESCs by single cell RNA-seq analysis is useful to characterize each differentiated stage [84].

As shown above, cell sorting of mesodermal progenitor cells, mesenchymal precursor cells, or myogenic cells is a powerful tool to obtain pure myogenic populations from differentiated pluripotent cells. Sakurai et al. have been able to induce PDGFR*α*(+)Flk-1(−) mesodermal progenitor cells by FACS followed by myogenic differentiation [85]. Chang et al. and Mizuno et al. have been able to sort SMC-2.6(+) myogenic cells from mouse ESCs/iPSCs [86, 87]. These SMC-2.6(+) myogenic cells were successfully engrafted into mouse regenerating skeletal muscle. However, this SMC-2.6 antibody only recognizes mouse myogenic cells but not human myogenic cells [86, 88]. Therefore, Borchin et al. have shown that hiPSC-derived myogenic cells differentiated into c-met(+)CXCR4(+)ACHR(+) cells, displaying that over 95% of sorted cells are Pax7(+) myogenic cells [72]. Taken together, current myogenic induction protocols utilizing small molecules and growth factors, with or without myogenic transcription factors, have been largely improved in the last 5 years. It is crucial to standardize the induction protocols in the near future to obtain sufficient myogenic cell conversion from pluripotent stem cells.

## 7. Epigenetic Myogenic Memory in Myoblast-Derived iPSCs

Recent work demonstrated that cells inherit a stable genetic program partly through various epigenetic marks, such as DNA methylation and histone modifications. This cellular memory needs to be erased during genetic reprogramming, and the cellular program reverted to that of an earlier developmental stage [16, 22, 89]. However, iPSCs retaining an epigenetic memory of their origin can readily differentiate into their original tissues [16–19, 90–100]. This phenomenon becomes a double-edged sword for the reprogramming process since the retention of epigenetic memory may reduce the quality of pluripotency while increasing the differentiation efficiency into their original tissues. DNA methylation levels are relatively low in the pluripotent stem cells compared to the high levels of DNA methylation seen in somatic cells [101]. Global DNA demethylation is required for the reprogramming process [102]. In the context of these observations, recent work demonstrates that activation-induced cytidine deaminase AID/AICDA contributing to the DNA demethylation can stabilize stem-cell phenotypes by removing epigenetic memory of pluripotent genes. This directly deaminates 5-methylcytosine in concert with base-excision repair to exchange cytosine in genomic DNA [103]. MicroRNA-155 has been identified as a key player for the retention of epigenetic memory during in vitro differentiation of hematopoietic progenitor cell-derived iPSCs toward hematopoietic progenitors [104]. iPSCs that maintained high levels of miR-155 expression tend to differentiate into the original somatic population more efficiently.

**Figure 3:**
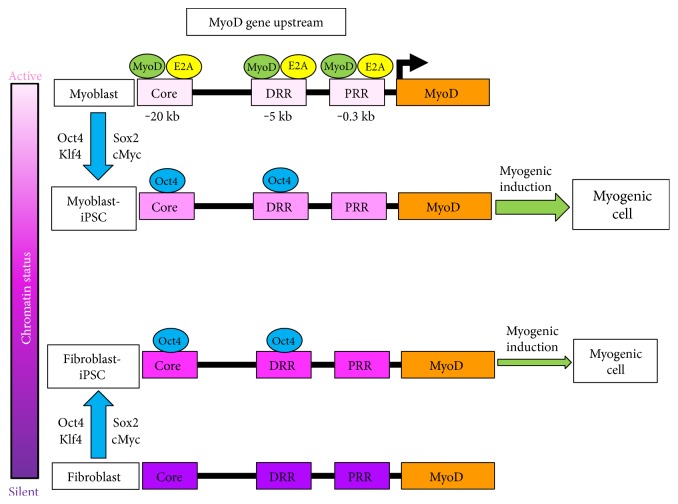
Schematic model for chromatin status of myoblast versus fibroblast-derived iPSCs for myogenic induction. In myoblasts, MyoD binds to the two MyoD enhancers (core and DRR) and promoter (PRR), and histone marks show the open chromatin state characteristic. During iPSC reprogramming via expression of Oct4, Sox3, Klf4, and cMyc, exogenous Oct4 binds to both MyoD enhancers which may lead to the bivalent state characteristic of pluripotent stem cells. In fibroblast, both MyoD enhancers and promoter show the closed chromatin state characteristic. During iPSC reprogramming, exogenous Oct4 binds to both MyoD enhancers which may lead to the bivalent state characteristic of pluripotent stem cells. However, myoblast-derived iPSCs may maintain the more open bivalent state characteristic, and thus, myogenic conversion efficiency is increased upon induction.

**Figure 4:**
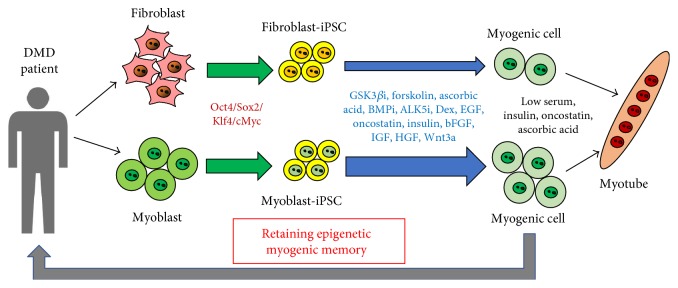
Myogenic cells induced from myoblast-derived iPSCs for DMD therapy. DMD patient-derived fibroblasts or myoblasts will be reprogramed into iPSCs by reprogramming factors (Oct4, Sox3, Klf4, and cMyc). These fibroblast- and myoblast-derived iPSCs will be induced to myogenic cells via combinatory small molecules and factors such as GSK3*β*i, forskolin, ascorbic acid, BMPi, ALK5i, Dex, EGF, oncostatin, insulin, bFGF, IGF, HGF, and Wnt3a. These iPSC-derived myogenic cells will be used for autologous cell therapy. Myoblast-derived iPSCs maintain epigenetic myogenic memory.

Recently, we generated murine skeletal muscle cell-derived iPSCs (myoblast-derived iPSCs) [23] and compared the efficiency of differentiation of myogenic progenitor cells between myoblast-derived iPSCs and fibroblast-derived iPSCs. After EB cultures, more satellite cell/myogenic progenitor cell differentiation occurred in myoblast-derived iPSCs than that in fibroblast-derived-iPSCs (unpublished observation and Figure 3), suggesting that myoblast-derived iPSCs are potential myogenic and satellite cell sources for DMD and other muscular dystrophy therapies (Figure 4). We also noticed that *MyoD* gene suppression by Oct4 is required for reprogramming in myoblasts to produce iPSCs (Figure 3) [23]. During overexpression of Oct4, Oct4 first binds to the Oct4 consensus sequence located in two MyoD enhancers (a core enhancer and distal regulatory region) [105–107] preceding occupancy at the promoter in myoblasts in order to suppress MyoD gene expression. Interestingly, Oct4 binding to the MyoD core enhancer allows for establishment of a bivalent state in MyoD promoter as a poised state, marked by active (H3K4me3) and repressive (H3K27me3) modifications in fibroblasts, one of the characteristics of stem cells (Figure 3) [23, 108]. It should be investigated whether the similar bivalent state is also established in Oct4-expressing myoblasts during reprogramming process from myoblasts to pluripotent stem cells. It remains to be elucidated whether Oct4-mediated myogenic repression only relies on repression of MyoD expression or is just a general phenomenon of functional antagonism between Oct4 and MyoD on activation of muscle genes. Nevertheless, myoblast-derived iPSCs will enable us to produce an unlimited number of myogenic cells, including satellite cells that could form the basis of novel treatments for DMD and other muscular dystrophies (Figure 4).

## 8. Conclusions

There are pros and cons of transgene-free small molecule-mediated myogenic induction protocols. In the transgene-mediated induction protocols, integration of the transgene in the host genome may lead to risk for insertional mutagenesis. To circumvent this issue, there is an obvious advantage for transgene-free induction protocols. Some key molecules such as Wnt, FGF, and BMP have used signaling pathways to induce myogenic differentiation of ES/iPSCs. However, these molecules are also involved in induction of other types of cell lineages, which makes it difficult for ES/iPSCs to induce pure myogenic cell populations in vitro. By contrast, transgene-mediated myogenic induction is able to dictate desired specific cell lineages. In any case, it is necessary to intensively investigate these myogenic induction protocols for the efficient and safe stem cell therapy for patients.

For skeletal muscle diseases, patient-derived hiPSCs, which possess the ability to differentiate into myogenic progenitor cells followed by myotubes, can be a useful tool for drug screening and personalized medicine in clinical practice. However, there are still limitations for utilizing hiPSC-derived myogenic cells for regenerative medicine. For cell-based transplantation therapies such as a clinical situation, animal-free defined medium is essential for stem cell culture and skeletal muscle cell differentiation. Therefore, such animal-free defined medium needs to be established for optimal myogenic differentiation from hiPSCs. Gene correction in DMD patient iPSCs by TALENs and CRISPR-Cas9 systems are promising therapeutic approaches for stem cell transplantation. However, there are still problems for DNA-editing-mediated stem cell therapy such as safety and efficacy. Since iPSC-derived differentiated myotubes do not proliferate, they are not suited for cell transplantation. Therefore, a proper culture method needs to be established for hiPSCs in order to maintain cells in proliferating the myogenic precursor cell stage in vitro in order to expand cells to large quantities of transplantable cells for DMD and other muscular dystrophies. For other issues, it is essential to establish methods to separate ES/iPSC-derived pure skeletal muscle precursor cells from other cell types for safe stem cell therapy that excludes tumorigenic risks of contamination with undifferentiated cells. In the near future, these obstacles will be taken away for more efficient and safe stem cell therapy for DMD and other muscular dystrophies.

## Figures and Tables

**Table 1:** Myogenic induction by overexpression of transgenes.

Authors	Year	Journals	Refs	Species	Cell types	Transgenes	Transgene systems	Remarks
Dekel et al.	1992	New Biol	[109]	Mouse	ESC	MyoD	Electroporation	EB culture
Rohwedel et al.	1995	Exp Cell res	[110]	Mouse	ESC	M-Twist	Transfection	EB culture
Prelle et al.	2000	Biochem Biophys Res Commun	[111]	Mouse	ESC	IGF2	Electroporation	EB culture
Myer et al.	2001	Dev Biol	[112]	Mouse	ESC	MyoD, myogenin	Electroporation	*Myogenin^−/−^* EB culture
Sumariwalla et al.	2001	Genesis	[113]	Mouse	ESC	MyoD, myogenin, MRF4	Electroporation	*Myogenin^−/−^* EB culture
Caron et al.	2005	Oncogene	[114]	Mouse	ESC	HMGA2/T	Transfection	EB culture
Kamochi	2006	Transplantation	[115]	Mouse	ESC	IGF2	Transfection	2D culture
Ozasa et al.	2007	Biochem Biophys Res Commun	[50]	Mouse	ESC	MyoD	Tet-Off system	2D culture
Darabi et al.	2008	Nat Med	[15]	Mouse	ESC	Pax3	Tet-ON system in integrated gene	EB culture, PDGFR*α*(+) Flk-1(−) cell sorting
Craft et al.	2008	Stem Cells	[116]	Mouse	ESC	Pax3, MyoD	Herpes simplex virus	EB culture
Warren et al.	2010	Cell Stem Cell	[51]	Human	iPSC	MyoD	mRNA transfection	EB culture
Meier-Stiegen et al.	2010	PLoS One	[117]	Mouse	ESC	Notch-IC-ERT	TMX-ERT system, electroporation	EB culture
Darabi et al.	2011	Stem Cells	[58]	Mouse	ESC	Pax7	Tet-ON system in integrated gene	EB culture, bFGF, PDGFR*α*(+) Flk-1(−) cell sorting
Iacovino et al.	2011	Stem Cells	[118]	Mouse, human	ESC	Myf5	Tet-ON system in integrated gene, Tet-ON-lentiviral vector	EB culture
Thoma et al.	2012	Cell Reprogram	[119]	Mouse	ESC	MyoD	TMX-induction system in transfection	EB culture
Goudenege et al.	2012	Mol Ther	[120]	Human	ESC	MyoD	Adenoviral vector	EB culture
Rao et al.	2012	Stem Cell Rev	[121]	Human	ESC	MyoD	Tet-On system in lentiviral vector	2D culture
Tedesco et al.	2012	Sci Transl Med	[80]	Human	iPSC	MyoD-ERT	TMX-ERT system, lentiviral vector	2D culture, induction of limb-girdle muscular dystrophy 2D, and DMD patient iPSC for mesoangioblast-like cells
Darabi et al.	2012	Cell Stem Cell	[59]	Human	ESC/iPSC	Pax7	Tet-ON system in lentiviral vector	EB culture, Pax7(+) cell sorting
Tanaka et al.	2013	PLoS One	[52]	Human	iPSC	MyoD	PiggyBac transposon-Tet-ON system	2D culture, Miyoshi myopathy patient hiPSC
Albini et al.	2013	Cell Rep	[54]	Human	ESC	MyoD + Baf60c	Lentiviral vector	Myosphere culture
Abujarour et al.	2014	Stem Cells Transl Med	[53]	Human	iPSC	MyoD	Tet-Off system in lentiviral vector	2D culture, DMD patient-derived hiPSC
Yasuno et al.	2014	Biochem Biophys Res Commun	[122]	Human	iPSC	MyoD	PiggyBac transposon-Tet-ON system	EB culture, carnitine palmitoyltransferase II deficiency patient hiPSC
Albini et al.	2014	J Vis Exp	[123]	Human	ESC	MyoD + Baf60c	Lentiviral vector	Myosphere culture
Li et al.	2015	Stem Cell Reports	[57]	Human	iPSC	MyoD	PiggyBac transposon-Tet-ON system	2D culture, DMD patient hiPSCs for gene correction by TALEN and CRISPR-Cas9
Maffioletti et al.	2015	Nat Protoc	[124]	Human	ESC/iPSC	MyoD-ERT	TMX-ERT system, lentiviral vector	2D culture, induction of limb-girdle muscular dystrophy 2D, and DMD patient iPSC for mesoangioblast-like cells
Shoji et al.	2015	Sci Rep	[56]	Human	iPSC	MyoD	PiggyBac transposon-Tet-ON system	2D culture, DMD patient hiPSCs for exon skipping
Dixon et al.	2016	Proc Natl Acad Sci U S A.	[125]	Human	ESC	MyoD	GAG-binding motif for cell penetrating peptide	2D culture
Shoji et al.	2016	Methods Mol Biol	[126]	Human	iPSC	MyoD	PiggyBac transposon-Tet-ON system	2D culture
Akiyama et al.	2016	Development	[127]	Human	iPSC	MyoD + JMJD3	PiggyBac transposon-Tet-ON system	2D culture
Magli et al.	2016	Methods Mol Biol	[128]	Mouse	ESC	Pax3	Tet-ON system in integrated gene	EB culture, PDGFR*α*(+) Flk-1(−) cell sorting

**Table 2:** Myogenic induction without transgenes.

Authors	Year	Refs	Journals	Species	Cell types	Factors	Remarks
Zhuang et al.	1992	[129]	Proc Natl Acad Sci U S A	Mouse	ESC		*E2A^−/−^* EB culture
Dinsmore et al.	1996	[130]	Cell Transplant	Mouse	ESC	RA, DMSO	EB culture of androgenetic and parthenogenetic ESC
Rohwedel et al.	1998	[131]	Exp Cell Res	Mouse	ESC	LiCl	EB culture
Barberi et al.	2005	[82]	PLoS Med	Human	ESC	OP9 and C2C12 coculture	CD73(+) MSC sorting
Barberi et al.	2007	[83]	Nat Med	Human	ESC	OP9 coculture, insulin	CD73(+) MSC sorting, NCAM(+) cell sorting
Sakurai et al.	2008	[85]	Stem Cells	Mouse	ESC		2D culture, PDGFR*α*(+) Flk-1(−) cell sorting
Sasaki et al.	2008	[132]	Differentiation	Mouse	ESC	Spermine	EB culture
Chang et al.	2009	[86]	FASEB J	Mouse	ESC		EB culture, SM/C-2.6(+) cell sorting
Sakurai et al.	2009	[68]	Stem Cell Res	Mouse	ESC	LiCl, BMP4	2D culture, PDGFR*α*(+) E-cadherin(low) cell sorting
Mizuno et al.	2010	[87]	FASEB J	Mouse	iPSC		EB culture, SM/C-2.6(+) cell sorting
Teng et al.	2010	[133]	J Cell Biochem	Human	ESC	TGF*β* inhibitor	*GNE^−/−^* EB culture
Awaya et al.	2012	[134]	PLoS One	Human	ESC/iPSC		EB culture
Sakurai et al.	2012	[135]	PLoS One	Mouse, human	ESC	LiCl, BMP4, Activin A	2D culture
Kuraitis et al.	2012	[136]	Eur Cell Mater	Mouse	ESC	sLeX-collagen matrices	EB culture
Xu et al.	2013	[70]	Cell	Human	iPSC	GSK3*β* inhibitor, bFGF, forskolin	EB culture
Leung et al.	2013	[137]	Biomacromolecules	Human	ESC	Chitosan-polycaprolactone (C-PCL) nanofibers + Wnt3a	2D culture
Borchin et al.	2013	[72]	Stem Cell Reports	Human	iPSC	GSK3*β* inhibitor	2D culture, c-met(+) cell sorting
Hosoyama et al.	2013	[75]	Stem Cells Transl Med	Human	ESC/iPSC	bFGF, EGF	EZ sphere culture
Hwang et al.	2014	[69]	Sci Rep	Mouse	ESC	Wnt3a	2D culture, PDGFR*α*(+) cell sorting
Shelton et al.	2014	[73]	Cell Reports	Mouse, human	ESC/iPSC	GSK3*β* inhibitor, BMP, VEGF, Inhibin*β*, bFGF	EB culture
Chal et al.	2015	[76]	Nat Biotechnol	Mouse, human	ESC/iPSC	GSK3*β* inhibitor, BMP inhibitor	2D culture
Chal et al.	2016	[77]	Nat Protoc	Human	iPSC	GSK3*β* inhibitor, BMP inhibitor, bFGF, HGF, IGF1	2D culture
Caron et al.	2016	[74]	Stem Cell Transl Med	Human	ESC	GSK3*β* inhibitor, Ascorbic acid, Alk5 inhibitor, Dex, EGF, insulin	2D culture
